# Evaluating the Hydrogen Evolution Reaction Activity of Colloidally Prepared PtSe_2_ and PtTe_2_ Catalysts in an Alkaline Medium

**DOI:** 10.1002/open.202400146

**Published:** 2024-07-23

**Authors:** Lineo F. Mxakaza, Victor Mashindi, Cebisa E. Linganiso, Nosipho Moloto, Zikhona N. Tetana

**Affiliations:** ^1^ Molecular Science Institute School of Chemistry University of the Witwatersrand Private Bag 3 Wits 2050 South Africa; ^2^ DSI/NRF Centre of Excellence in Strong Materials University of the Witwatersrand Private Bag 3 Wits 2050 South Africa; ^3^ Institute for Nanotechnology and Water Sustainability College of Science Engineering and Technology University of South Africa, Florida 1709 Johannesburg South Africa

**Keywords:** hydrogen evolution reaction, Pt dichalcogenides, PtSe_2_, PtTe_2_

## Abstract

The hydrogen evolution reaction (HER) in alkaline electrolytes using transition metal dichalcogenides is a research area that is not tapped into. Alkaline HER (${2H}_{2}O+2{e\;}^{-}\to H_{2}+{OH}^{-}\;$ ) is harder to achieve relative to acidic HER ($H^{+}+2{e\;}^{-}\to \;H_{2}$ ), this is attributed to the additional water dissociation step that occurs in basic HER to generate H^+^ ions. In fact, for most catalysts, their HER activity decreases tremendously when the electrolyte is changed from acidic to basic conditions. Platinum dichalcogenides, PtX_2_ (X=S, Se, Te), are an interesting member of transition metal dichalcogenides (TMDs) as these show an immense hybridization of the Pt d orbitals and chalcogen p orbitals because of closely correlated orbital energies. The trend in electronic properties of these materials changes drastically as the chalcogen is changed, with PtS_2_ reported to exhibit semi‐conductor properties, PtSe_2_ is semi‐metallic or semi‐conductive, depending on the number of layers, while PtTe_2_ is metallic. The effect of varying the chalcogen atom on the HER activity of Pt dichalcogenides will be studied. Pt dichalcogenides have previously been prepared by direct high‐temperature chalcogen deposition of Pt substrate and evaluated as electrocatalysts for HER in H_2_SO_4_. The previously employed synthesis procedures for PtX_2_ limit these compounds′ mass production and post‐synthesis treatment. In this study, we demonstrated, for the first time the preparation of PtSe_2_ and PtTe_2_ by colloidal synthesis. Colloidal synthesis offers the possibility of large‐scale synthesis of materials and affords the employment of the colloids at various concentrations in ink formulation. The electrochemical HER results acquired in 1 M KOH indicate that PtTe_2_ has a superior HER catalytic activity to PtSe_2_. A potential of 108 mV for PtTe_2_ and 161 mV for PtSe_2_ is required to produce a current density of −10 mA cm^−2^ from these catalysts. PtTe_2_ has a low Tafel slope of 79 mVdec^−1^, indicating faster HER kinetics on PtTe_2_. Nonetheless, the stability of these catalysts in an alkaline medium needs to be improved to render them excellent HER electrocatalysts.

## Graphical abstract

Colloidal synthesis of Pt dichalcogenides in an organic solvent was carried out at 320°C, resulting in the formation of crystalline structures that can be used to split water under alkaline conditions to generate hydrogen. PtTe_2_ has a comparable HER catalytic activity to commercial Pt/C.

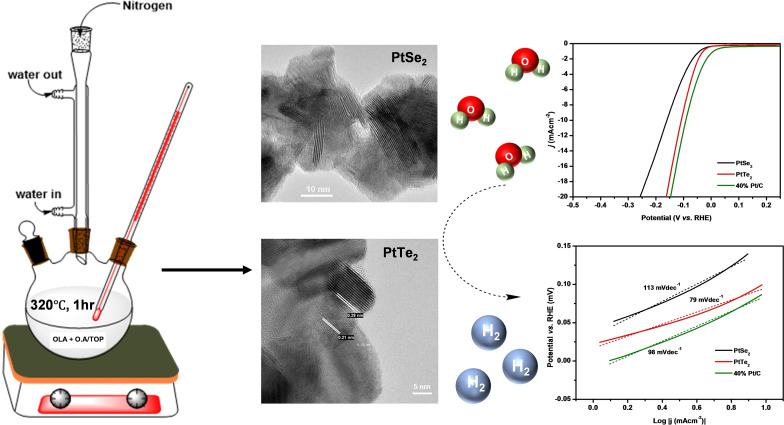

## 1 Introduction

Hydrogen is a critical aspect of various industrial applications^[1]^ and is a fundamental energy carrier.^[2]^ One of the environmentally benign hydrogen production procedures is water splitting. Water splitting comprises the oxygen evolution and hydrogen evolution reaction halves. Ultimately, these gases (oxygen and hydrogen) can be utilized as feedstock in fuel cells to produce energy.^[3]^ Water, however, is a poor electrical conductor and to improve its electron conductivity, the pH of water can be altered through the addition of electrolytes (acid/base). A vast amount of research has been focussed on the hydrogen evolution reaction (HER) in an acid medium.^[4]^ This is purely because acid HER is relatively easy to achieve compared to HER in an alkaline medium. The HER process in a basic medium follows the three reactions shown below (Eqn. 1–Eqn. 3) and conforms to either the Volmer‐Heyrovsky or the Volmer‐Tafel mechanisms.^[5]^ In the Volmer step, the water molecule is dissociated first into OH^−^ ions and hydrogen atoms (H) that are adsorbed to the catalyst surface (M), labeled as MH. For the H_2_ generation, either the adsorbed hydrogen atoms interact to generate H_2_ and the free metal surface (Tafel), or the reduction of water and the MH interaction yields H_2_ and OH^−^ ions (Heyrosky).^[6]^ The water dissociation step and the large overpotential of HER in an alkaline medium, result in a relatively slow and hard‐to‐achieve alkaline HER. Consequently, the HER efficiency of most electrocatalysts is reported to decline with increasing electrolyte pH, even for platinum.^[7]^ Nevertheless, alkaline water electrolysis offers sustainable industrialization owing to the prolonged stability of electrodes in alkaline electrolytes relative to acidic media and eliminates the need for expensive proton exchange membranes.^[8]^

(1) $$Volmer:\;{2H}_{2}O+2{e\;}^{-}+\;M\;\to {OH}^{-}+\;MH\;\left(adsorbed\right)$$

(2) $$Heyrovsky:\;MH\;\left(\mathrm{adsorbed}\right)+\;H_{2}O+{e\;}^{-}\to M+\;H_{2}+\;{OH}^{-}$$

(3) $$Tafel:\;\mathrm{MH}+\mathrm{MH}\;\to M+\;H_{2}$$

To achieve lower overpotentials, improved efficiencies, and enhanced stability of electrode materials in an alkaline electrolyte, the adsorption energy of water and hydrogen on the active sites must be moderate. Simultaneously, a low attraction of hydroxyl ions towards the catalyst surface is required to facilitate the reaction in an alkaline medium.^[9]^ High adsorption of hydroxyl ions on the electrode can lead to electrode poisoning, wherein the active sites become blocked by OH^−^ ions.^[5]^ Pt is a rare earth element and therefore limits the commercialization and sustainability of Pt electrode‐based devices. The overall quantity of the Pt consumed can be reduced by doping poor‐performing catalysts with traces of Pt, or through coordination of Pt with other atoms to generate Pt compounds such as nitrides, phosphides, oxides, and chalcogenides.[^10^, ^11^] Development of Pt compounds such as hydroxides, chalcogenides, and oxides has been reported as one way of reducing the energy required for the water dissociation step.^[12]^ Platinum dichalcogenides, (PtX_2_, X=S, Se, Te) are an interesting class of transition metal dichalcogenides. Firstly, the Pt−X covalent bond in PtX_2_ compounds intensifies down the chalcogen group. Secondly, the traditional van der Waals interlayer interactions observed in other transition metal dichalcogenides (TMDs) are not observed in PtX_2_ compounds, rather X−Pt−X layer interactions are covalent because of the degree of hybridization between the chalcogen bonding p orbitals and Pt bonding d orbitals.[^13^, ^14^] These characteristics render platinum dichalcogenides properties to have relatively more drastic chalcogen and layer‐dependent properties. For instance, a few layers of PtSe_2_ result in a semi‐conductive material, whereas multi‐layered PtSe_2_ is semi‐metallic.^[15]^

Pt dichalcogenides have previously been prepared by thermally assisted conversion coupled with chemical vapor deposition, mechanical exfoliation, and molecular beam epitaxy.[^14^, ^16^, ^17^] The shortcomings of these synthesis procedures are the limited control of the size of the prepared nanosheets, and the difficulty in upscaling the procedures. In this study, colloidal synthesis, which is a high‐temperature crystal formation in an organic solvent will be carried out to address these limitations. The major advantage of employing colloidal synthesis for nanomaterial preparation is the ability to control the size, composition, and morphology of the nanoparticles by manipulating the reaction parameters.[^18^, ^19^, ^20^] Reaction parameters that can be manipulated and optimized in colloidal synthesis include the surfactant, precursor type, concentration and ratio, temperature, and reaction time.^[21]^ The platinum dichalcogenides will be evaluated as alkaline electrocatalysts. The primary goal is to minimize platinum usage by using a Pt compound instead of the traditional all‐platinum electrode.

## Experimental Section

### Chemicals and Materials

Oleylamine (C_18_H_37_N, OLA, 70 %, Sigma‐Aldrich), oleic acid (C_18_H_34_O_2_, O.A, 90 %, Sigma‐Aldrich), platinum (II) acetylacetonate (C_10_H_4_O_4_Pt, Pt(AcAc)_2_, 97 %, Sigma‐Aldrich), selenium powder (Se, 99.5 %, Sigma‐Aldrich), trioctylphosphine (C_24_H_5_P, TOP, 97 %, Sigma‐Aldrich), platinum (IV) chloride (PtCl_4_, 99.9 %, Sigma‐Aldrich), tellurium powder (Te, 99.8 %, Sigma‐Aldrich), toluene (C_6_H_5_CH_3_, 99 %), Nafion perfluorinated resin solution (5 weight %), isopropanol (C_3_H_8_O, 99 %, Sigma‐Aldrich), ultra‐pure water, ethanol (C_3_H_5_OH, 99.9 %, Sigma‐Aldrich), 40 % commercial Pt/C (Tanaka Kikinzoku Kogyo), graphite rod (Metrohm), Ag/AgCl auxiliary reference electrode (Metrohm), glassy carbon electrode (GC, Metrohm), potassium hydroxide (KOH, 85 %, Sigma‐Aldrich) and carbon black (Johnson and Mathey).

### Colloidal Synthesis of PtSe_2_ and PtTe_2_

The colloidal synthesis of Pt dichalcogenides has not been reported before. The reaction process, either hot injection or heat‐up reaction in various high‐boiling solvents, reaction times, and solvents used in the synthesis were optimized for the preparation of PtSe_2_ and PtTe_2_. The optimum conditions were found to be slightly different for each of the materials.

### Colloidal Synthesis of PtSe_2_

A hot injection synthesis procedure of platinum and selenium precursors in a hot organic solvent was employed to prepare PtSe_2_. Typically, a 1 : 1 v/v (total volume=10 mL) ratio of OLA and O. A was added to a three‐neck glass round bottom flask connected to a condenser with a constant nitrogen flow. The solvent mixture was then heated to 320 °C using an electro mantle with a maximum heating temperature of 380 °C equipped with a magnetic stirrer. The solution was systematically stirred to ensure uniform heat distribution. At this point, a mixture of Pt(AcAc)_2_, (0.244 mol) and elemental selenium (0.488 mol) was injected. The solvent solution immediately changed color from pale yellow to black. The reaction temperature was then maintained at 320 °C for 1 h, followed by cooling to 60 °C and then adding toluene to precipitate out the nanoparticles. The PtSe_2_ nanoparticles were ultimately isolated by centrifuging the obtained solution at 8000 rpm for 10 min for three cycles, then dried in the fume hood.

### Colloidal Synthesis of PtTe_2_

Similarly, a hot inject colloidal synthesis procedure was employed for the preparation of PtTe_2_. 8 mL of OLA and 2 mL of TOP were added to a three‐neck round bottom flask, connected to the Schlenk line. The system was saturated with nitrogen gas while stirring and heating the solution to 320 °C, using a magnetic stirrer equipped electro mantle. Steady stirring of the solution was carried out to ensure uniform heat distribution. In a vial, PtCl_4_ (0.270 mol) and Te (0.540 mol) powders were mixed and injected into the hot pale‐yellow solution which immediately turned black. The solution temperature was then maintained at 320 °C for 1 h. The reaction solution temperature was then allowed to decline to 60 °C and toluene was added to precipitate out the PtTe_2_ nanoparticles. The cool solution was then placed in centrifuge tubes, transferred to the centrifuge, and rotated for 10 min at 8000 rpm. A black product was dried in the fume hood, crushed, and analyzed.

### Characterization Techniques Employed for the Platinum Dichalcogenides

The Raman spectra were recorded on a Horiba Scientific MacroRam Raman Spectrometer equipped with a 785 nm laser, Olympus BX41 microscope, and the crystallographic parameters of the nanoparticles were analyzed by X‐ray diffraction (XRD) using a Bruker D2 Phaser Powder X‐ray diffractometer fitted with a Cu Kα X‐ray source (1.54 nm). Measurements were taken over 2θ angle range 10–90° in steps of 0.026° with a step time of 5 s at ambient conditions. High‐Resolution Transmission Electron Microscopy (HRTEM, using JEOL JEM 2100 at 200 kV equipped with a Thermo Fischer detector for EDS analysis), and X‐ray photoelectron spectroscopy (XPS, Thermo Scientific ESEAlab 250Xi using a 300 W Monochromatic Al kα (1486.7 eV) X‐ray beam with a diameter of 900 μm and 20 eV pass energy) characterization techniques were employed to ascertain the morphology and the elemental oxidation states in the nanomaterials. The HER activity and the resistance of the prepared catalysts were determined through electrochemical measurements carried out using the Biologic SP 300, rotating disk potentiostat. A three‐electrode cell set‐up comprising of graphite rod (counter electrode), Ag/AgCl (reference electrode), and modified glassy carbon working electrode (glassy carbon coated with an ink of the prepared catalysts, 0.196 cm^2^) all immersed in 1 M KOH (pH=14) electrolyte were used for the measurements. A 2.55 mL dissolving solution containing 1 mL isopropanol, 1.5 mL ultrapure water, and 50 μL of Nafion was used to disperse 3.0–3.2 mg of the PtSe_2_ and PtTe_2_ catalysts and 1.0–1.2 mg of carbon black. The ink mixture was sonicated and then 10 μL of the ink was drop‐casted on a pre‐cleaned 0.196 cm^2^ glassy carbon electrode (previously cleaned by scrubbing on a 1.0 μm and 0.5 μm alumina paste and rinsing with ultra‐pure water). The ink was dried by rotating the glassy carbon at 250 rpm for approximately 45 min. The calculated catalyst loading was 80–88 μg cm^−2^. The Pt, Se, and Te quantities in the ink were determined by ICP‐OES conducted on a Thermo Icap 6500 ICP‐OES. The height and roughness of the working electrode were studied using the Nanosurf Core Atomic Force Microscope (CoreAFM), equipped with an Isostage 300 controller. All contact mode AFM scans were acquired under ambient conditions using areas of 10 mm×10 mm using ContAl‐G silicon cantilevers with resonant frequency of 13 kHz and a force constant of 0.2 N/m. The images were acquired at a resolution of 256×256 points and processed using the Nanosurf CoreAFM software. The AFM sample was prepared by drop casting 10 μL of the PtSe_2_ and PtTe_2_ inks onto a 1 cm×1 cm glassy carbon electrode and dried by rotating at 250 rpm. Linear sweep voltammetry scans of the electrode at 5 mV/s were obtained by applying a potential (−2 V to 0.5 V) to the working electrode while rotating the working electrode at 1600 rpm. The electrochemical impedance spectroscopy (EIS) was carried out by applying potential obtained from the LSV curve at onset potential at a frequency range of 0.1 Hz–100 kHz while rotating at 1600 rpm. The stability of the electrodes was determined by running the LSV before and after 1000 CV cycles, and by studying the current density response over time using current‐time chronoamperometry (CA) studies carried out at η_−10_. Temperature‐dependent LSV studies were done by connecting the cell to a water bath fitted with a small pump. LSV curves were then obtained when the electrolyte temperature was set at 298 K, 308 K, 318 K, 328 K, and 338 K in the potential window of −0.2 V to 0.5 V. All measurements were performed in a 150 mL electrochemical cell conjugated with argon gas. Before plotting, the potential was corrected against the reference hydrogen electrode (RHE) using Eqn. 4 and Eq. 5.

(4)

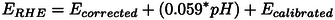

(5) $$E_{corrected}=E_{measured}-\left(iR\right)$$

Where *i* is the measured current, R is solution resistance and E_calibrated_ is the Ag/AgCl potential.

## 2 Results and Discussion

### 2.1 Characterization of the Platinum Dichalcogenides

**Figure 1:**
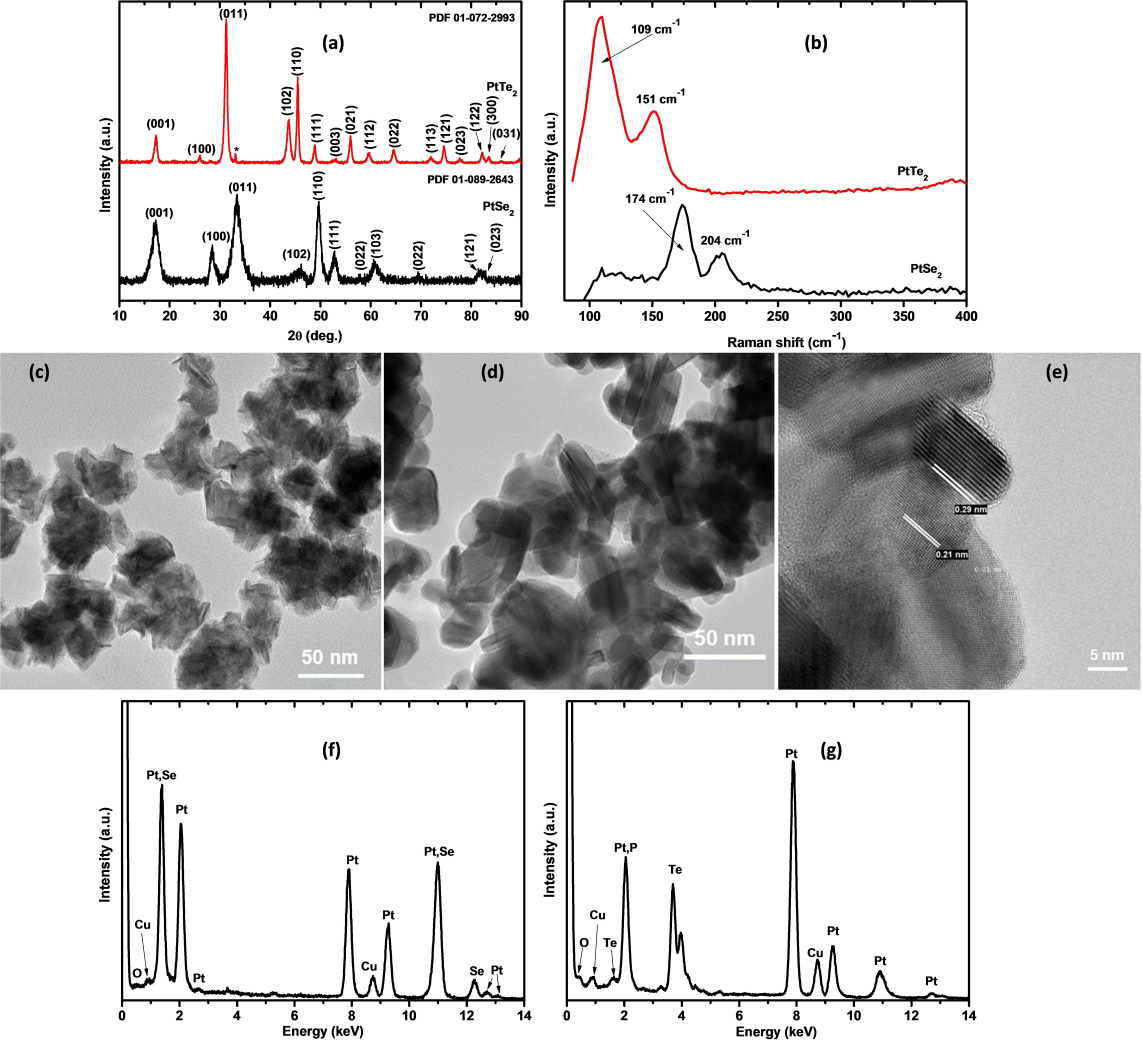
(a) XRD patterns and (b) Raman spectra of PtSe_2_ (black) and PtTe_2_ (red). TEM images of (c) PtSe_2_, (d) PtTe_2,_ and (e) HRTEM image of PtTe_2_ and the corresponding EDS spectra of (f) PtSe_2_ and (g) PtTe_2_.

The crystallinity, phase purity, and identification of the prepared nanoparticles were carried out through powder XRD analysis. Figure 1(a) shows the XRD patterns of PtSe_2_ and PtTe_2_ from 2θ=10° to 2θ=90°. The prepared PtSe_2_ was matched to a hexagonal syn‐sudovikovite crystal structure with a space group of P‐3 m1 and lattice parameters a=3.72, b=3.72, and c=5.06. PtTe_2_, on the other hand, was indexed to hexagonal moncheite with a space group of P‐3 m1 and lattice parameters a=4.01, b=4.01, and c=5.2. These materials share the same space group, P‐3 m1, and exhibit identical crystal symmetry, signifying similar overall structural characteristics. Nonetheless, distinctions in lattice parameters set these materials apart, influencing crucial aspects of their crystal structures. Lattice parameters dictate the size and shape of the unit cell, impacting both unit cell volume and, consequently, material density. The arrangement of atoms within the unit cell, as reflected in these parameters, plays a significant role in determining various physical properties, including electronic and catalytic behavior. While the space group provides insights into the broader symmetry, variations in lattice parameters may indicate discrete atomic positions within the unit cell, potentially influencing phase transitions in response to external conditions. Consequently, it suggests that PtSe_2_ and PtTe_2_ could potentially exhibit different catalytic activities. Figure 1(b) shows the Raman spectra of PtSe_2_ and PtTe_2_. The Raman active modes of PtSe_2_ are reported to appear at 180 cm^−1^ and 210 cm^−1^ because of the in‐plane and out‐of‐plane Pt−Se vibrations, respectively.^[22]^ The observed peaks in the prepared PtSe_2_ are at 174 cm^−1^ (E_g_) and 204 cm^−1^ (A_1g_), comparable to the literature. These vibration modes occur at lower frequencies in PtTe_2_. The PtTe_2_ E_g_ vibration mode is observed at 109 cm^−1^ while the A_1g_ mode occurs at 151 cm^−1^, in good agreement with previous studies.^[23]^

TEM analysis (Figure 1(c) and (d)) of both PtSe_2_ and PtTe_2_ was carried out to determine the 2D structure of the materials. Both materials resulted in the formation of irregularly arranged and poly‐dispersed clusters of sheet‐like materials. The two sheet‐like structures were distinctively different with PtSe_2_ showing flower‐like morphologies while PtTe_2_ showed plate‐like structures. Furthermore, the high‐resolution image of PtTe_2_ in Figure 1(e), showed the presence of two lattice fringes with d‐spacing of 0.29 and 0.21 nm corresponding to (011) and (102) crystal planes of moncheite, respectively. The EDS spectra in Figure 1(f) and (g) show that PtSe_2_ comprised mainly Pt and Se atoms, whereas the PtTe_2_ spectrum showed the presence of Pt and Te as major components. The Cu and O are attributed to the copper grid and surface oxidation.

**Figure 2:**
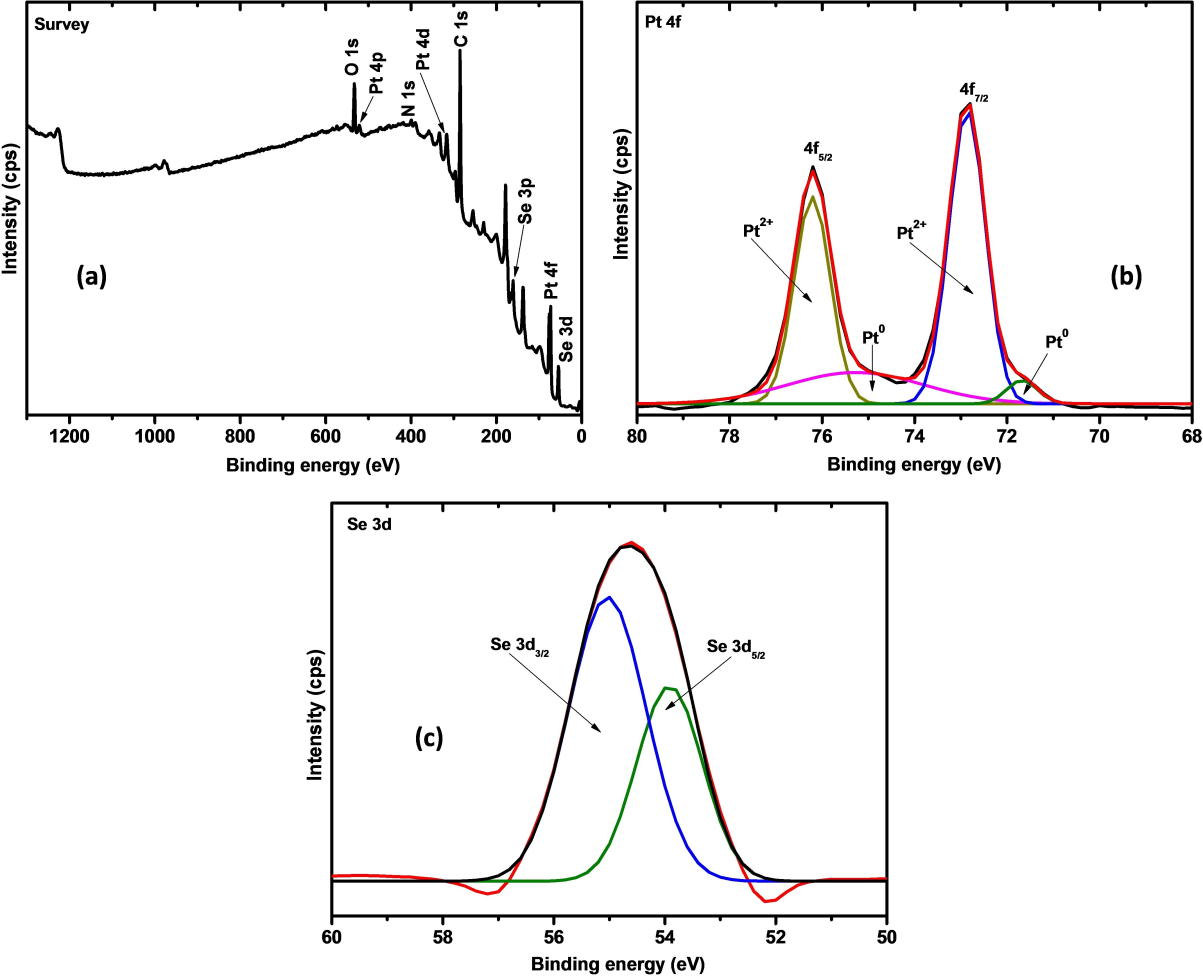
(a) XPS survey spectrum of PtSe_2_ and the high‐resolution spectra of (b) platinum and (c) selenium elements.

The XPS analysis of PtSe_2_ was carried out to determine the identity and oxidation states of the elements in PtSe_2_. The survey spectrum of PtSe_2_, shown in Figure 2(a), shows that the material contained predominantly platinum, selenium, carbon, oxygen, and some traces of nitrogen. Carbon, oxygen, and nitrogen are attributed to the capping agents used in the synthesis of PtSe_2_. The high‐resolution spectrum of Pt in Figure 2(b) shows the presence of two types of platinum oxidation states. The peak pair at ∼ 76.2 eV and 72.9 eV are attributed to Pt^2+^ whereas the peaks at 75.2 eV and 71.9 eV are attributed to elemental platinum (Pt^0^), consistent with previous reports.[^16^, ^22^] We can conclude that there is no presence of oxidized Pt within the PtSe_2_ samples due to the absence of the PtO_2_ peak at ∼ 73.9 and ∼ 77.3 eV.^[24]^ The high‐resolution scan of Se in Figure 2(c) shows a broad peak that is deconvoluted into two peaks at 55.0 eV and 53.9 eV. These peaks are consistent with the Se^2−^ peaks observed for various metal selenide materials.[^16^, ^25^] These observations suggest that colloidal synthesis of PtSe_2_ in a mixture of oleylamine and oleic acid resulted in the formation of PtSe_2_, with traces of unreacted Pt.

**Figure 3:**
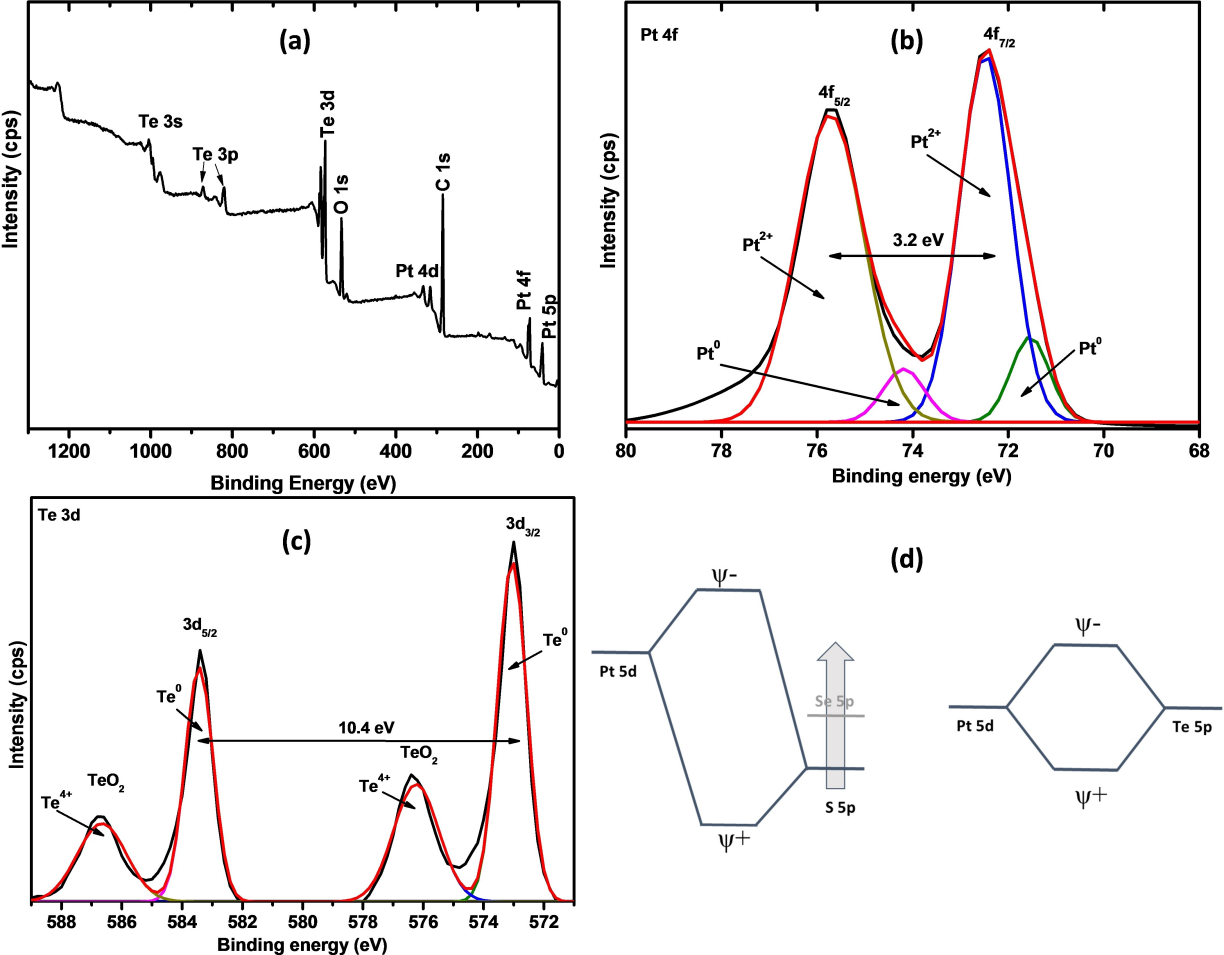
(a) XPS survey, (b) Pt and (c) Te high‐resolution XPS spectra of PtTe_2,_ and (d) the trend in bonding configuration of platinum dichalcogenides.^[31]^

The XPS survey spectrum of PtTe_2_ nanoparticles in Figure 3 shows four intense peaks at 570–589 eV, 534 eV, 284 eV, and 70–78 eV attributed to platinum, carbon, oxygen, and tellurium, respectively. This observation is consistent with other XPS measurements carried out on PtTe_2_.[^26^, ^27^] The Pt high‐resolution scan (Figure 3(b)) displays two dominant peaks at 75.7 eV and 72.5 eV which are characteristic of Pt^2+^.^[16]^ The two low area peak pairs at 74.2 and 71.5 eV are attributed to Pt^0^.[^24^, ^28^] These results are consistent with the observations made by Yang et al.,^[29]^ Supriya et al.,^[26]^ and Chia et al.^[27]^ However, Yang and Supriya highlight vaguely that the peaks are due to Pt in PtTe_2_ and do not indicate the exact oxidation state of Pt. On the other hand, Chia and co‐workers argue that the obtained oxidation states of Pt and Te in PtTe_2_ (Pt=+2, Te=+4) diverge from the anticipated oxidation states (Pt=+4, Te=−2). The XPS high‐resolution spectrum of tellurium shown in Figure 3(c) illustrates the presence of two Te oxidation states in the PtTe_2_ material. The peak at 573 eV and its corresponding shadow peak at 583 eV with a peak‐to‐peak spacing of 10 eV is due to elemental Te (0 oxidation state).^[30]^ On the other hand, the peaks at 576 and 586 eV occur because of the presence of oxidized tellurium in the prepared material. The oxidation state of Te in its oxidized form is +4.[^26^, ^31^] These observations suggest the presence of only Te (0) and Te (+4) in the PtTe_2_. Due to the strong covalent nature of the Pt−Te bond, its electronic structure is better described as Pt metal possessing an oxidation state within the range of 0 to +2 rather than +4 while the Te chalcogen adopts an oxidation state of about 0 instead of −2.^[30]^ The bonding orbital energies of sulfur are much lower in energy to Pt d orbitals than Se and Te, depicted in Figure 3(d). As one progresses down the chalcogen group, the chalcogen p orbital energy‐Pt d orbital energy difference diminishes.^[31]^

### 2.2 Hydrogen Evolution Reaction Properties of Platinum Dichalcogenides

**Figure 4:**
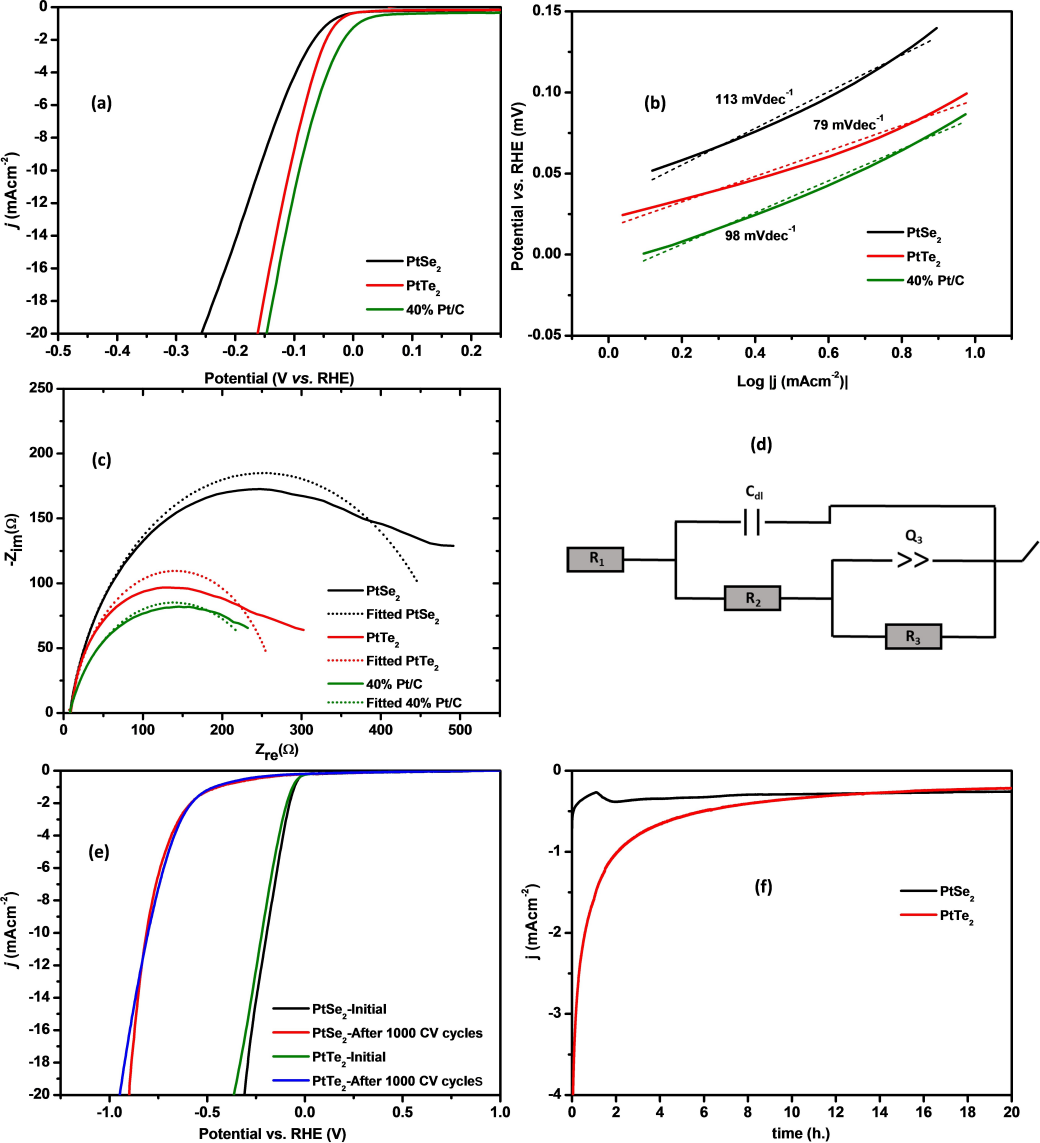
Comparison of (a) HER polarization curve, (b) Tafel plots, (c) experimental and fitted Nyquist plots of PtSe_2_ and PtTe_2_ to 40 weight % commercial Pt/C catalyst, (d) is the electrochemical cell model that was fitted to the EIS data, and (e) is the LSV curves of PtSe_2_ and PtTe_2_ after a stabilized CV cycle and after 1000 CV cycles (CV cycles obtained between −1 V and 0.5 V at 5 mVs^−1^) and (f) is the CA curves obtained at onset potentials of the respective catalysts.

The HER activity of Pt dichalcogenides was investigated by comparing the following performance indicators: onset potential, overpotential, Tafel slope, exchange current density (*j*_o_) as well as charge transfer resistance (R_ct_) obtained from the polarization curves, Tafel plot, and Nyquist plot, all shown in Table 1. The plots were obtained by running the electrochemical measurements in 1 M KOH at room temperature using a scan rate of 5 mVs^−1^ and rotating the working electrode (PtX_2_ where X=Se; Te deposited on GC) at 1600 rpm. Figure 4(a) depicts the LSV of the prepared catalysts. The graph clearly illustrates that PtTe_2_ (onset potential=28 mV, η_−10_=108 mV) has a comparable HER activity to the commercial 40 % Pt/C (onset potential=7 mV, η_−10_=93 mV), whereas PtSe_2_ has a low HER activity in the same electrolyte. These results show a similar trend to previous studies carried out in an acidic medium on the same catalyst materials.[^26^, ^27^] Chia et al. found out that PtTe_2_ prepared by chemical vapor deposition of chalcogen on the Pt surface is a better‐performing HER catalyst than PtSe_2_.^[27]^ The observed onset potential and η_−10_ of PtTe_2_ in KOH is extensively lower than that of both PtSe_2_ and PtTe_2_ in an acidic medium, suggesting that PtTe_2_ is a good catalyst for HER in a basic medium. HER in the basic medium is relatively harder to achieve as opposed to acidic medium HER since for hydrogen to be produced in KOH, water needs to be broken down in hydroxyl and hydroxide ions. This process is more energy intensive than the combination of hydroxyl ions to produce hydrogen gas in acidic medium.[^32^, ^33^]

**Table 1:** Comparison of performance indicators for HER on various catalysts.

HER Catalyst	Electrolyte	Onset η/mV	η_−10_ mA cm^−2^/mV	b/mVdec^−1^	J/mAcm^−2^	Rct/Ω	Reference
PtSe_2_	1 M KOH	44	161	113±4	0.513	500	This work
PtTe_2_	1 M KOH	28	108	79±3	0.378	269	This work
40 % Pt/C	1 M KOH	7	93	98±2	1.364	266	This work
MoSe_2_	1 M KOH	~290	331	137	–	~60	[34]
MoS_2_	1 M KOH	350	450	105	–	14	[35]
Ni_3_S_2_	1 M KOH	–	171	132	–	10	[36]

The production of hydrogen from either acidic or basic electrolytes is a three‐step process that involves hydrogen adsorption on the catalyst surface (Volmer step), followed by either electrochemical (Heyrovsky step) or chemical desorption of hydrogen gas (Tafel step). Generally, HER on a catalytic surface occurs through the Volmer‐Heyrovsky mechanism or Volmer‐Tafel mechanism, with the Heyrovsky obtained if the Tafel slope is very large.^[4]^ It can then be assumed that HER on PtSe_2_, PtTe_2,_ and Pt/C follows the Volmer‐Heyrovsky mechanism. A low Tafel slope is also indicative of fast reaction kinetics and a low energy barrier. Figure 4(b) shows the comparison of Tafel plots of the catalysts. The relatively low Tafel slope (79 mVdec^−1^) of PtTe_2_ suggests that PtTe_2_ possesses higher HER activity compared to PtSe_2_ (113 mVdec^−1^). The Tafel plot can then be used to extrapolate the minimum current that can be produced by the catalysts when the potential is zero. This parameter is the *j*_o_ and is used to ascertain the catalytic activity of the catalysts. The higher the *j*_o_ value is, the more catalytic the electrode material is. PtSe_2_ shows a higher *j*_o_ of 0.513 mAcm^−2^, while *j*_o_ of PtTe_2_ is 0.378 mAcm^−2^. This means that more current is produced from the PtSe_2_ catalysts at equilibrium. The EIS spectra shown in Figure 4(c) were fitted to an electrochemical cell depicted in Figure 4(d). R_ct_ values obtained from this fit indicate the resistance of the electrode surface to electron flow, that is R_ct_ signifies how conductive the electrode is. The R_ct_ values for PtSe_2_, PtTe_2,_ and Pt/C were found to be 500 Ω, 269 Ω, and 266 Ω, respectively. This suggests that the charge transfer occurs faster on both PtTe_2_ and 40 % Pt/C than on PtSe_2_. PtTe_2_ is deemed to exhibit metallic properties while PtSe_2_ shows semi‐metallic properties. The conductivity of PtTe_2_, by its nature, is expected to surpass that of PtSe_2_.

The prolonged stability of the electrode material was determined by running LSV before and after 1000 CV scans (Figure 4(e)). Although the catalysts show a high catalytic activity in the basic medium, their stability is quite poor. This is evidenced by an increased η_−10_ and onset potential (taken at −1 mAcm^−2^) after 1000 CV scans, indicating that the catalytic activity of the catalysts reduces after prolonged exposure to the electrolyte. CA studies (Figure 4(f)), where the current response was measured over 20 hours show a very low current response, less than 0.5 mAcm^−2^, for both catalysts. The current response for PtSe_2_ only stabilises after 2 h while the current response for PtTe_2_ declines gradually over the 20 h period. The HER performance parameters of the catalysts prepared in this study and those of previously evaluated 2D TMDs are summarized in Table 1. Generally, PtSe_2_ and PtTe_2_ show a small onset and η_−10_, and comparable Tafel slope to the other catalysts. The R_ct_ values of PtSe_2_ and PtTe_2_ are relatively high. This suggests that electron transfer between the external circuit and the KOH electrolyte is harder to achieve when using PtSe_2_ and PtTe_2_.

**Figure 5:**
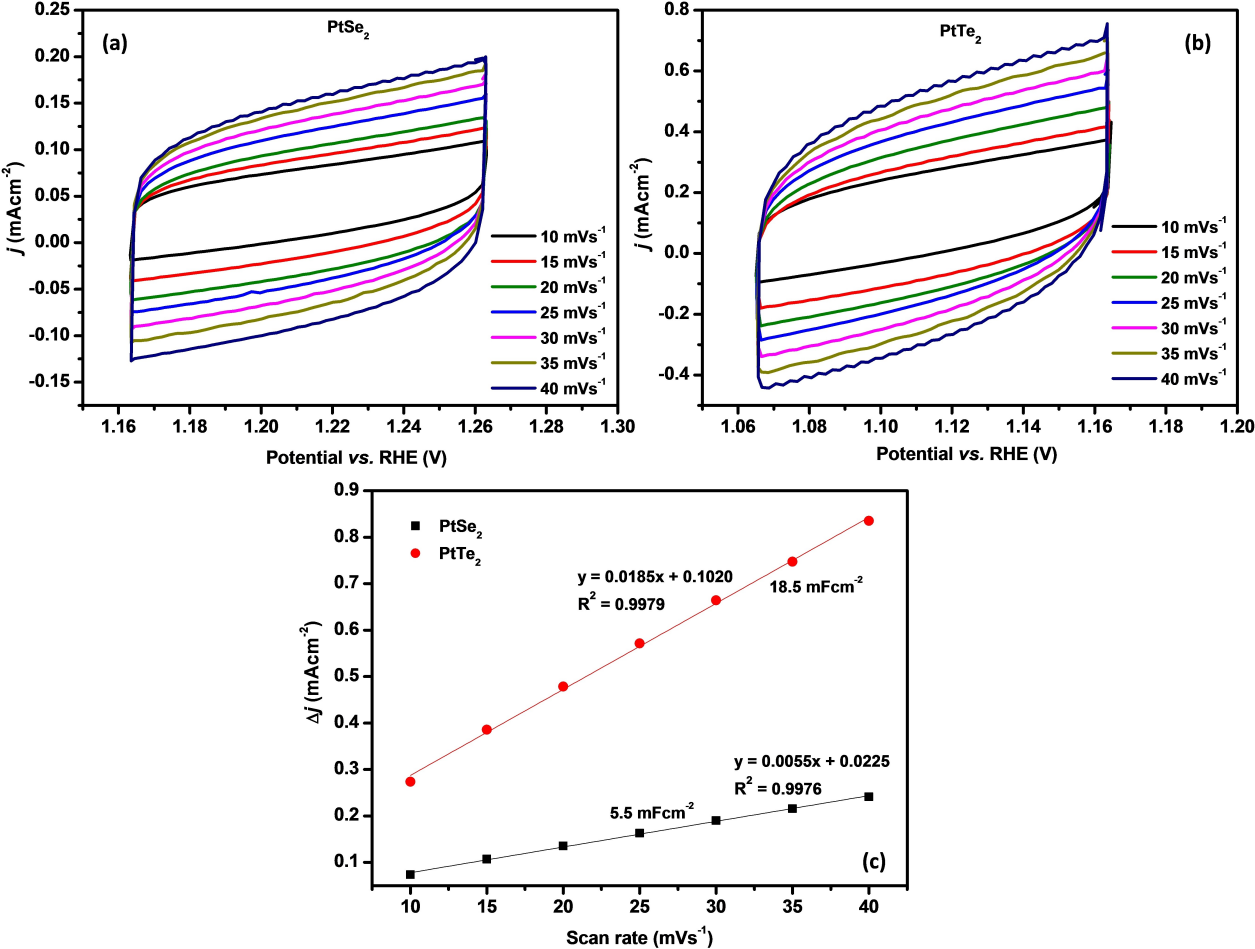
Non‐Faradaic CV curves of (a) PtSe_2_, (b) PtTe_2_ at increasing scan rates, and (c) the double layer capacitance measurements.

Several catalyst properties influence the electrochemical reactions of the catalyst, one of which is the electrochemical surface area (ECSA). ECSA can be estimated from the double layer capacitance (C_dl_) and specific capacitance (C_s_) related by equation 5 below. Firstly, the change in current density as the scan rate is increased from 10 mVs^−1^ to 40 mVs^−1^ was studied in the non‐Faradaic current response region. (Figure 5(a) for PtSe_2_ and Figure 5(b) for PtTe_2_). A proportional relationship between scan rate and current density was observed.

(6) $$ECSA=\;\frac{C_{dl}}{C_{s}}$$

C_dl_ was then determined from the slope of the linearized plot of change in current density (▵j, j_a_–j_c_), against scan rate shown in Figure 5(c). The C_dl_ of PtTe_2_ was found to be higher than that of PtSe_2_. This observation can help us understand why PtTe_2_ has a superior HER activity over PtSe_2_. The ECSA of PtTe_2_ was then calculated to be 462.5, three times higher than that of PtSe_2_ (137.5).

**Figure 6:**
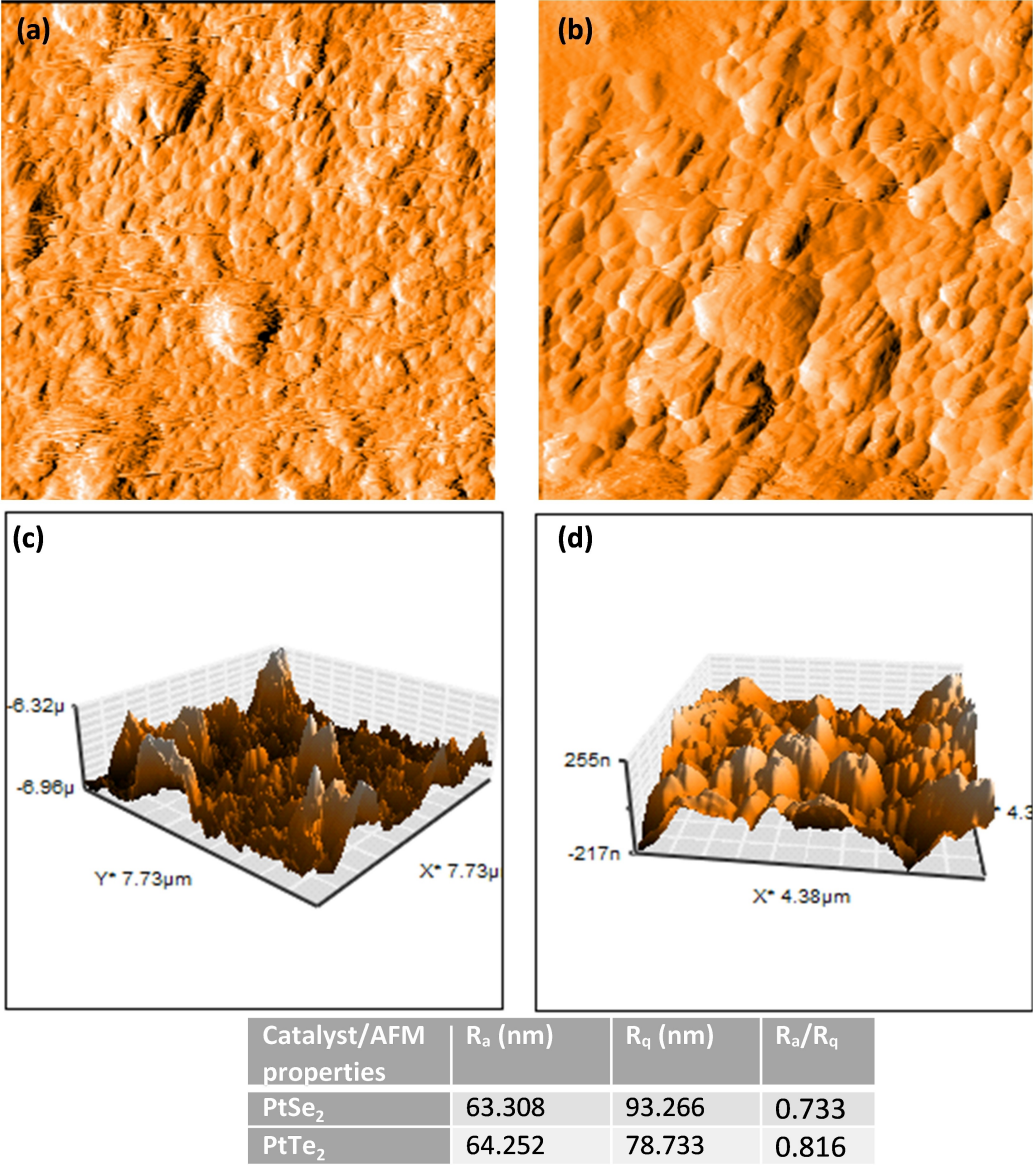
AFM surface topology images of (a) PtSe_2_ and (b) PtTe_2_ inks and the height profile of (c) PtSe_2_ and (d) PtTe_2_.

To better understand the catalyst surfaces and their role in the HER, we studied the morphology of the working electrode. The topology images in Figure 6(a) and (b) indicate the presence of clustered grains with differing heights and lengths. The surface roughness, average roughness divided by root mean square,^[37]^ ($\frac{R_{a}}{R_{q}})$ of PtSe_2_ was calculated as 0.733 while that of PtTe_2_ was 0.816. This signifies that the surface of PtTe_2_ electrode was rougher than PtSe_2_, potentially introducing more grooves that increase the surface area.

**Figure 7:**
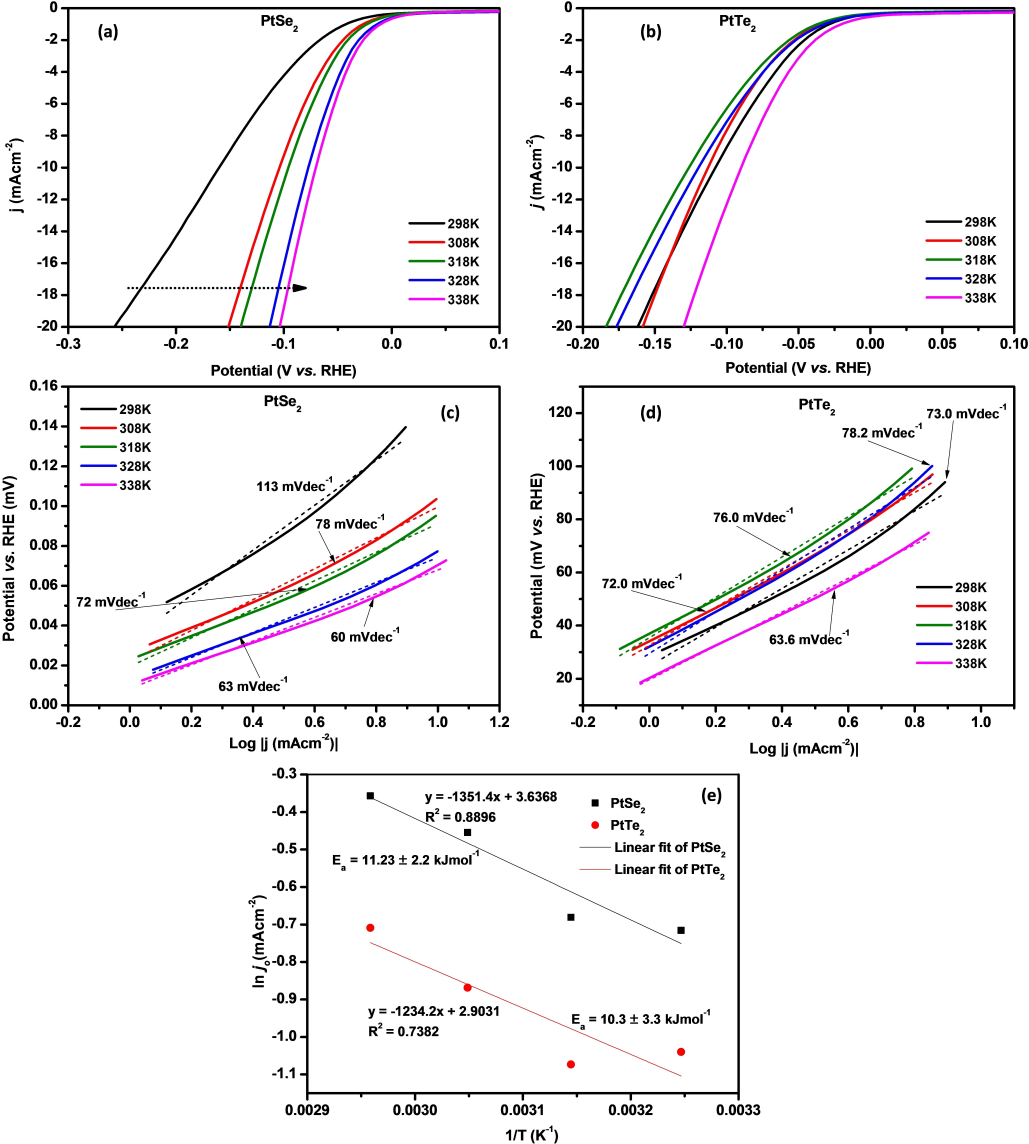
LSV curves of (a) PtSe_2_ and (b) PtTe_2_ obtained at increasing electrolyte temperature, corresponding Tafel plots of (c) PtSe_2_ and (d) PtTe_2_ and, (e) Arrhenius plots for PtSe_2_ and PtTe_2_.

The HER activity of electrocatalysts is reported to change with the electrolyte temperature. To study this effect, LSV polarization curves of both PtSe_2_ and PtTe_2_ were obtained at 298 K, 308 K, 318 K, 328 K, and 338 K. The corresponding Tafel slopes from the LSV curves were also obtained. The onset potential decreases from 45.0 mV at 298 K to 11.5 mV at 338 K, while the η_−10_ changes from 161.2 mV to 70.3 mV at the same temperatures for PtSe_2_ (Figure 7(a)). Likewise, the onset potential and the η_−10_ of PtTe_2_ decreased from 29.9 mV and 107.4 mV, at 298 K to 21.9 mV and 89.8 mV at 338 K, respectively. (Figure 7(b)), Figure 7(c) & (d) shows the Tafel slopes at the respective temperatures. The slope for both catalysts is reduced upon increasing the electrolyte temperature, this suggests faster HER kinetics at elevated temperatures. The Tafel plots were then linearized to get *j*_o_. This is the current produced when there is no energy input in the system. The minimum energy required to facilitate the HER on the catalysts, referred to as activation energy (E_a_) can then be obtained from the plot of the inverse of temperature against ln *J*_o_ (Figure 7(e)) deduced from the Arrhenius equation shown below (Eqn. 6). The slope of this plot is then related to $\frac{E_{a}}{R}$ . From this, the E_a_ values obtained for PtSe_2_ was 11.2 kJ mol^−1^, with that of PtTe_2_ slightly lower at 10.3 kJ mol^−1^. Both these values are lower than E_a_ values reported by various authors.[^38^, ^39^] Lower E_a_ values signify faster reaction initiation, enhanced reaction efficiency, improved kinetics, and reduced overpotential. This further suggests that the energy required for the HER on PtX_2_ catalysts is low, as such, hydrogen is easily produced from these catalysts’ surfaces.

(7) $$k=Ae^{\frac{{-E}_{a}}{RT}}$$

where *k* is the kinetic rate constant, $E_{a}\;$ is the activation energy, A is the collision frequency factor, R is the gas rate constant and T is the temperature in Kelvin.

The exact amount of Pt in the catalysts and the actual Pt loading was determined by inductively coupled plasma‐optical emission spectroscopy (ICP‐OES) and the results are summarised in Table 2. The mass percent concentration of Pt in PtSe_2_ was determined to be 2.55 % and slightly lower at 2.23 % in PtTe_2_ sample. Thefore, the Pt loading on the PtSe_2_ coated GC was 33.5 μg/cm^2^ and 27.8 μg/cm^2^ on PtTe_2_ coated GC electrodes. Despite having low Pt loading, PtTe_2_ displays a better alkaline HER activity than PtSe_2_. The carbon black loading, which was used to improve the conductivity of the catalysts, was calculated and obtained as 24 μg/cm^2^.

**Table 2:** ICP‐OES mass percentage concentration of the catalysts and corresponding Pt loading.

Catalyst	Mass percent concentration (% m/m)			Pt loading (μg/cm^2^)
	Pt	Se	Te	
PtSe_2_	2.55	2.36	–	33.54
PtTe_2_	2.23	–	2.85	27.84

## 3 Conclusions

This work highlights the hot‐injection colloidal synthesis of PtSe_2_ and PtTe_2_ in a mixture of OLA and TOP/O . A and the evaluation of their catalytic activity towards HER. The key performance indicators of an HER catalyst material are onset potential, Tafel slope, exchange current density, charge transfer resistance, and stability in the electrolyte. Generally, the electrochemical properties of PtTe_2_ were found to be identical to those of commercial Pt/C. This can be attributed to the more metallic nature of PtTe_2_. The better performance of PtTe_2_ relative to PtSe_2_ can be attributed to its high ECSA and roughness. Additionally, the activation energy of PtTe_2_ was found to be slightly lower than that of PtSe_2_, implying that less energy is required to initiate the HER on the PtTe_2_ catalyst. This is even though the Pt loading when using PtTe_2_ was 27.8 μg/cm^2^ whereas Pt loading was higher when using PtSe_2_ (33.5 μg/cm^2^) as the HER catalyst. As such, PtTe_2_ is concluded to be a promising HER catalyst in a basic medium. This observation suggests that good HER activities can be achieved by developing Pt‐based compounds, such as Pt dichalcogenides, with low Pt loading. Although both PtSe_2_ and PtTe_2_ are suitable candidates for basic medium HER, their stability in the KOH electrolyte is poor and needs to be improved. Strategies such as coalescing or supporting the platinum dichalcogenides on carbon compounds such as graphene or carbon nanotubes can be considered to improve the overall stability of the compounds.

## Conflict of Interests

The authors declare no conflict of interest.

4

## Data Availability

The data that support the findings of this study are available from the corresponding author upon reasonable request.
